# Prevalence of talon cusps in Jordanian permanent teeth: a radiographic study

**DOI:** 10.1186/1472-6831-10-6

**Published:** 2010-04-20

**Authors:** Abed Al-Hadi M Hamasha, Rima A Safadi

**Affiliations:** 1 Department of Preventive Dentistry, Faculty of Dentistry, Jordan University of Science and Technology, Irbid, Jordan; 2 Department of Oral Medicine and Surgery, Faculty of Dentistry, Jordan University of Science and Technology, Irbid, Jordan

## Abstract

**Background:**

The aim of the study is to investigate the prevalence of talon cusps in a sample of Jordanians dental patients and their distribution among different types of teeth.

**Methods:**

The data were collected from radiographic examination of 3,024 periapical films showing 9,377 teeth from a random sample of 1,660 patients. A tooth was considered having talon cusp if there was a V-shape radiopaque structure superimposed the tooth structure.

**Results:**

Talon cusps were detected in 52 teeth (tooth prevalence = 0.55%). Maxillary canines were the most commonly affected teeth (46% of cases), followed by maxillary lateral incisor teeth (39% of cases) and maxillary central incisors teeth (15% of cases). Teeth with talon cusps were found in 40 subjects (person prevalence = 2.4%). Bilateral talon cusps were seen in 12 patients.

**Conclusions:**

Attention should be paid to the presence of talon cusp and the treatment problems associated with it.

## Background

**Figure 1:**
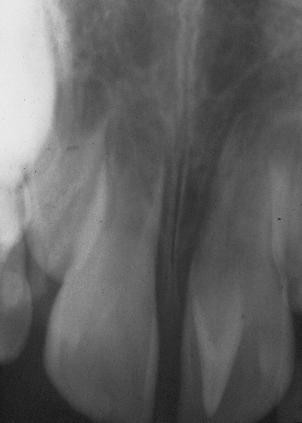
**Talon cusp radiograph**. Periapical radiograph of upper central incisor showing a V-shape structure superimposed over the normal image of the crown

Talon cusp is a prominent accessory cusp-like structure projecting from the cingulum area or cementoenamel junction (CEJ) of the maxillary or mandibular teeth in both primary and permanent dentition [1,2]. This projection was termed talon cusp because of it bear a resemblance to an eagle's talon in shape. This cusp normally presented in the palatal or occlusal surfaces of the teeth, however, there were some reported cases of talon cusps in labial surfaces of teeth [3,4]. To consider this projection as a talon cusp, it must extends at least one millimetre or more beyond CEJ [5] or half the distance from CEJ to the incisal edge [6] (Figure 1).

Hattab *et al*. [2] proposed a classification system for talon cusps, on the basis of the degree of cusp formation and extension: Type 1 (talon) refers to a morphologically well-delineated additional cusp that projects prominently from the palatal (or facial) surface of a primary or permanent anterior tooth and extends to at least half the distance from the CEJ to the incisal edge. Type 2 (semi talon) refers to an additional cusp (≥ 1 mm) that extends to less than half the distance from the CEJ to the incisal edge. It may blend with the palatal surface or stand away from the rest of the crown. Type 3 (trace talon) is an enlarged or prominent cingulum that may appear as conical, bifid, or tubercle [2].

The aetiology of the condition is unknown. However it was hypothesized to be due to combination of genetic and environmental factors [7]. Developmentally, talon cusps may be resulted from outfolding of inner enamel epithelial cells and focal hyperplasia of the peripheral cells of mesenchymal dental papilla [7]. Talon cups can occur as isolated conditions or associated with other dental anomalies including bifid cingula, peg-shaped incisors, dens invaginatus, shovel-shaped incisors and exaggerated cups of Carabelli [8].

Radiographically, the appearance of a talon cusp is similar to that of normal tooth material, presenting with radiopaque enamel and dentin with or without extension of pulpal tissues [9]. Typically, talon cusp looks like a V-shape structure superimposed over the normal image of the crown. When the tooth is unerupted, a radiographic talon cusp may resemble mesiodens, compound odontoma, supernumerary tooth or a dens invaginatus [10].

The clinical problems associated with the presence of talon cusps include stagnation of food, caries, periapical lesions, irritation of tongue during speech and mastication, other soft tissue irritation, breast feeding problems, compromised aesthetics, occlusal interference which may lead to accidental cusp fracture, displacement of the affected tooth, temporomandibular joint pain, and periodontal problems because of excessive occlusal force [11].

The prevalence of talon cusps was mistakenly estimated by calculating the number of talon cusps reported in the literature. Danker *et al*.[12] reviewed a number of 108 reported talon cusps as case-reports in the literature between the years of 1970 and 1995. The authors indicated that about 7.7% of the cases were in permanent teeth and 20% of them were bilaterally distributed [12] About half of reported talons were in maxillary lateral incisors, about a third was in maxillary central incisors and only about 8% were in canines [12].

Studies that have addressed the prevalence of talon cusps in populations were scarce. The prevalence of talon cusps was reported as 0.6% in a Mexican [13], 2.5% in a Hungarian [14], 5.2% in a Malaysian [15] and 7.7% in an Indian populations [5]. No data were found to address the distribution of talon cusps among different tooth types in population studies.

The purpose of this study was to investigate the prevalence of talon cusps in a sample of Jordanians dental patients and their distribution among different type of teeth.

## Methods

An initial random sample of 2,111 dental records was selected from the Jordan University of Science and Technology's (JUST) dental archive. The sample frame consisted of 12,395 records for private dental patients and 1,800 records for university employees. The final sample used in this study was 1,660 dental records including a total number of 3,024 periapical radiographs showing 9,377 teeth. Selection of the subjects were based on the availability of radiographs, however a presetting criteria for having at least two comparable periapical radiographs for both side of jaw was set. This study was approved by JUST Ethics Committee (152/2006).

Two experienced examiners read all radiographs in a dark room with a 10-X magnifying lens and an X-ray viewer (Illuminator 5000, RP Beard Ltd., London, England). A tooth was considered having talon cusp if there was V-shape radiopaque structure superimposes the tooth structure. Each radiograph exhibit this criterion was re-examined carefully by both examiners twice and a combined decision was made to either consider the tooth is having talon cusp or not.

The examiners were calibrated by reading 100 radiographs separately, containing 10 different cases of talon cups before the investigation starts. The diagnosis given by both examiners were compared to their original diagnosis which resulted in 100% agreement.

The examined teeth from periapical radiographs were recorded on a data sheet as normal teeth or teeth with talon cusps. The observations were entered and analyzed using the computer program, SPSS 12. (SPSS Inc. Chicago, USA).

## Results

Of the teeth examined, 5,633 (60.1%) were for males and 3,744 (39.9%) were for females. Ages ranged between 18 and 69 years, with a mean age of 25.1 years (SD = 8.05).

Almost equal numbers of maxillary (4,713) and mandibular teeth (4,664) were examined. The number of individual tooth type was comparable so the per tooth prevalence of talon cusps was meaningful. Talon cusps were detected in 52 teeth out of a total of 9377 teeth to give a tooth prevalence of 0.55%. The prevalence of talon cusps among different tooth types was presented in Table 1. All 52 teeth exhibiting talon cusps were in the maxillary arch, so the maxillary teeth prevalence was 1.1%. Maxillary canines were most commonly affected teeth in the mouth (46% of cases), followed by maxillary lateral incisor teeth (39% of cases) and maxillary central incisors teeth (15% of cases). No talon cusps were detected in any other tooth types.

**Table 1:** The prevalence of talon cusps among different tooth types

Tooth type		No. of teeth examined	No. of teeth with talon cusps	Prevalence %
Maxillary	Central incisor	528	8	1.5
	
	Lateral Incisor	515	20	3.9
	
	Canine	427	24	5.6
	
	First premolar	509	0	0.0
	
	Second premolar	639	0	0.0
	
	First molar	783	0	0.0
	
	Second molar	704	0	0.0
	
	Third molar	608	0	0.0
	
	Subtotal	4713	52	1.1

Mandibular	All mandibular teeth	4664	0	0.0

Total		9377	52	0.55

Table 2 presents the distribution and prevalence of talon cusps according to the gender of patients. Males had more teeth with talon cusps (32 teeth) than females (20 teeth). The prevalence of talon cusp for males and females was 0.57% and 0.53%, respectively. However the difference was not statically significant using chi square test (χ^2 ^= 0.05, p = 0.47).

**Table 2:** Distribution of subjects with and without talon cusps among different gender.

Gender	No. of teeth (% of teeth)		Total
		
	With talon cusp*	Normal teeth	
Male	32 (0.57)	5601(99.43)	5633

Females	20 (0.53)	3724(99.47)	3744

Total	52 (0.55)	9325(99.45)	9377

* P-value = 0.47

The distribution of patients with talon cusps is presented in Table 3. Teeth with talon cusps were found in 40 subjects (26 males, 14 females) out of 1,660 subjects examined, thus the person prevalence was 2.4%. Bilateral talon cusps were seen in 12 patients (30%), whereas 28 patients (70%) of those with talon cusps exhibited unilateral talon cusps.

**Table 3:** Distribution of subjects with talon cups.

Subjects with:	Male	Female	Total
Talon cusps	26	14	40

None (All normal teeth)	971	649	1,620

Total	997	663	1,660

Percentage	2.61	2.11	2.41

Subjects with			

Double talon cusps	6	6	12

Single talon cusps	20	8	28

Total	26	14	40

Percentage of double talon cusps (out of those with talon cusps)	23.1	42.9	30.0

## Discussion

The present study was based on radiographic examination of periapical radiographs which were taken for a variety of purposes including full mouth dental screening and diagnosis of dental problems. Not all records were belonged to fully dentate patients nor all records contained full mouth periapical radiographs. In this study, approximately 2-3 periapical films per person were examined. This does not constitute a review of the whole mouth of the subjects examined. Despite the fact that diagnosis of dental anomalies based on radiographs solely without clinical examination might produce a false positive/or false negative diagnoses. However, talon cusps are easily detected radiographically as they typically present as V-shaped formations superimposed on the tooth. An exception to that is a type 3 trace talon cusp which cannot be detected in radiographic examination. This study investigated the presence of talon cusps in permanent teeth; no examination of permanent teeth in children was attempted. The results present the prevalence of talon cusps only in patients attended dental clinics at JUST. However, there is no reason to believe that dental patients are different from other Jordanian adults. No data was found to indicate genetic, social and geographic differences in the prevalence of talon cusps among other nations.

Talon cusps were found in 40 subjects out of 1,660 subjects examined. Of the subjects examined, a maximum of 528 (32%) had radiographs available of their maxillary anterior teeth. Therefore, the person prevalence of the condition (2.4%) was probably underestimated.

Most of the published studies dealing with talon cusps were case reports. Some of these studies estimated the prevalence of talon cusps by counting the number of cases reported in the literature with talon cusps. These reported cases were grouped into different age and gender groups [2,6,7,12]. The results obtained using this procedure should be taken with caution because it is not possible to report all cases of talon cusps present in population. Additionally, the design of these studies does not constitute a community base screening of the anomaly and the criteria and definition of the condition vary from one case reports to another.

The person prevalence of talon cusp in the present study was 2.4%. These results are comparable with what was reported in Hungry (2.5%), [14] but lower than what was reported for Indian (7.7%) [5] and Malaysian (5.5%) [15] populations. The variation in talon cusp prevalence could be explained by variation of the condition among different nations, or variation in the samples examined, or examination criteria.

In the present study the maxillary canines were the most commonly affected teeth with the condition, followed by the lateral incisors and central incisors. However, in the literature, maxillary lateral incisors were the most commonly affected teeth, followed by the central incisors and canines. This might be explained by that the sample used in this study might be different than that of other nations. This difference might also be due to the fact that our study is radiographic survey of radiographs from dental records. In the literature, the distribution of talon cusps among different types of teeth was based on the case-reports or case-series of the condition.

In the present study, the prevalence of talon cusps of the maxillary anterior teeth was 3.5%. About 2.4% of the subjects examined exhibited one or more talon cups. The present study is the first to report person prevalence of talon cusp and the distribution of anomaly among all tooth type. This will provide more information on the types of teeth that are more susceptible to this anomaly and need more attention during radiographic examination.

## Conclusions

Talon cup is uncommon dental anomaly. The availability of such data will allow anticipation of the percentage of teeth that might have technical difficulties associated with the endodontic treatment of such teeth. It will also facilitate the understanding of changes in occlusion and periodontal conditions associated with the anomaly. It will allow better communication with the patient regarding their unusual symptoms and their endodontic, prosthodontic, periodontic, restorative and cosmetic need.

## Competing interests

There are no competing interests (political, personal, religious, ideological, academic, intellectual, commercial or any other) to declare in relation to this manuscript.

## Authors' contributions

All work is the solely work of both authors of this study. Both authors read and approved the final version of the manuscript.

## Pre-publication history

The pre-publication history for this paper can be accessed here:

http://www.biomedcentral.com/1472-6831/10/6/prepub

## References

[B1] Peker I Alkurt MT Talon cusp: a case series Gen Dent 2009 57 524 527 19903646

[B2] Hattab FN Yassin OM al-Nimri KS Talon cusp in permanent dentition associated with other dental anomalies: review of literature and reports of seven cases ASDC J Dent Child 1996 63 368 376 8958353

[B3] Pomeroy E Labial talon cusps: a South American archaeological case in the deciduous dentition and review of a rare trait Br Dent J 2009 206 277 282 10.1038/sj.bdj.2009.168 19287429

[B4] Tulunoglu O Cankala DU Ozdemir RC Talon's cusp: report of four unusual cases J Indian Soc Pedod Prev Dent 2007 25 52 55 10.4103/0970-4388.31993 17456971

[B5] Chawla HS Tewari A Gopalakrishnan NS Talon cusp--a prevalence study J Indian Soc Pedod Prev Dent 1993 1 28 34 6595354

[B6] Davis PJ Brook AH The presentation of talon cusp: diagnosis, clinical features, associations and possible aetiology Br Dent J 1986 160 84 88 10.1038/sj.bdj.4805774 3456236

[B7] al-Omari MA Hattab FN Darwazeh AM Dummer PM Clinical problems associated with unusual cases of talon cusp Int Endod J 1999 32 183 90 10.1046/j.1365-2591.1999.00212.x 10530205

[B8] McNamara T Haeussler AM Keane J Facial talon cusps Int J Paediatr Dent 1997 7 259 262 10.1046/j.1365-263X.1997.00052.x 9482033

[B9] Lomcali G Hazar S Altinbulak H Talon cusp: report of five cases Quintessence Int 1994 25 431 433 7938433

[B10] Tsutsumi T Oguchi H Labial talon cusp in a child with incontinentia pigmenti achromians: case report Pediatr Dent 1991 13 236 237 1886829

[B11] Gungor HC Altay N Kaymaz FF Pulpal tissue in bilateral talon cusps of primary central incisors: report of a case Oral Surg Oral Med Oral Pathol Oral Radiol Endod 2000 89 231 235 10.1067/moe.2000.102877 10673662

[B12] Dankner E Harari D Rotstein I Dens evaginatus of anterior teeth. Literature review and radiographic survey of 15,000 teeth Oral Surg Oral Med Oral Pathol Oral Radiol Endod 1996 81 472 475 10.1016/S1079-2104(96)80027-8 8705596

[B13] Sedano HO Carreon Freyre I Garza de la Garza ML Gomar Franco CM Grimaldo Hernandez C Hernandez Montoya ME Hipp C Keenan KM Martinez Bravo J Medina López JA Clinical orodental abnormalities in Mexican children Oral Surg Oral Med Oral Pathol 1989 68 300 301 10.1016/0030-4220(89)90215-6 2671852

[B14] Mavrodisz K Rózsa N Budai M Soós A Pap I Tarján I Prevalence of accessory tooth cusps in a contemporary and ancestral Hungarian population Eur J Orthod 2007 29 166 169 10.1093/ejo/cjl084 17317866

[B15] Rusmah Meon Talon cusp in Malaysia Aust Dent J 1991 36 11 14 10.1111/j.1834-7819.1991.tb00801.x 2029226

